# Diagnostic value of leucine-rich alpha-2-glycoprotein 1 and calprotectin in acute appendicitis: a short review

**DOI:** 10.11613/BM.2025.030504

**Published:** 2025-10-15

**Authors:** Lea Gvozdanović, Željka Dragila, Luka Maršić, Denis Klapan, Željka Dujmić, Josip Samardžić, Zrinka Mihaljević, Višnja Nesek-Adam

**Affiliations:** 1 Emergency Department, General Hospital Našice, Našice, Croatia; 2 Faculty of Medicine, Josip Juraj Strossmayer University of Osijek, Osijek, Croatia; 3 Emergency Department, University Hospital Center Osijek, Osijek, Croatia; 4 Emergency Department, General Hospital “Dr. Josip Benčević”, Slavonski Brod, Croatia; 5 Faculty of Dental Medicine and Health Osijek, Josip Juraj Strossmayer University of Osijek, Osijek, Croatia; 6 Office of Quality Management, General Hospital “Dr. Josip Benčević”, Slavonski Brod, Croatia; 7 Department of Abdominal Surgery, General Hospital “Dr. Josip Benčević”, Slavonski Brod, Croatia; 8 Department of Physiology and Immunology, Faculty of Medicine, Josip Juraj Strossmayer University of Osijek, Osijek, Croatia; 9 Department of Anesthesiology, Clinical Hospital “Sveti Duh”, Zagreb, Croatia

**Keywords:** appendicitis, biomarkers, inflammation

## Abstract

Acute abdominal pain accounts for 7-10% of all emergency department visits. Appendicitis, being one of the leading causes of acute abdominal surgery, poses significant diagnostic challenges. Negative appendectomy rates can be as high as 40%, while complications occur in more than 90% if the diagnosis is missed during the initial examination. Therefore, more effective preoperative screening is required for patients with suspected appendicitis. Recent studies suggest that novel biomarkers, particularly leucine-rich alpha 2-glycoprotein and calprotectin, may improve the early and accurate diagnosis of acute appendicitis by demonstrating high specificity and sensitivity, respectively. Unlike C-reactive protein, the production of leucine-rich alpha 2-glycoprotein 1 and calprotectin takes place at the site of inflammation. As a result, their raised concentrations might be evident early in a disease, possibly before other common markers of inflammation start to rise. This literature review aims to assess the potential role of leucine-rich alpha 2-glycoprotein 1 and calprotectin as diagnostic biomarkers in patients with suspected acute appendicitis, acknowledging the need for additional data to fully assess their diagnostic accuracy.

## Graphical abstract

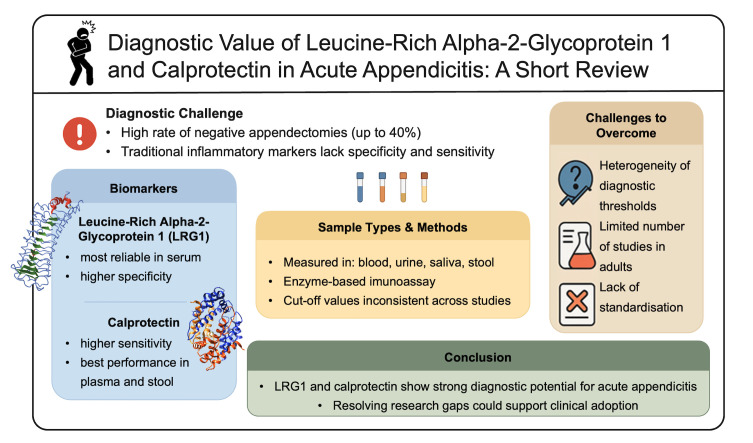

## Highlights

• The rate of negative appendectomies remains high, reaching up to 40%

• Leucine-rich alpha-2-glycoprotein 1 and calprotectin show promise for acute appendicitis diagnosis

• Leucine-rich alpha-2-glycoprotein 1 has higher specificity, while calprotectin improves sensitivity

• Combining both biomarkers with clinical scores may improve diagnostic accuracy

• Standardized cut-off values are needed for reliable biomarker-based diagnosis

## Introduction

Acute abdominal pain accounts for 7-10% of all emergency department visits (*1*). Acute appendicitis is one of the most common causes of non-traumatic lower abdominal pain and the most frequent surgical emergency in young adults. Its incidence has declined in high-income countries since the 1940s but remains notable, with 5.7-50 cases *per* 100,000 people annually and a peak between ages 10 and 30. Lifetime risk varies by region, ranging from 9% in the USA and 8% in Europe to 2% in Africa (*2*). However, accurately diagnosing the condition remains challenging. The clinical diagnosis is primarily based on medical history, physical examination including right lower quadrant tenderness, rebound pain, and guarding, as well as laboratory tests that reflect systemic inflammation. Despite their widespread use, commonly used markers such as white blood cell (WBC) count and C-reactive protein (CRP) are nonspecific and insufficient to reliably distinguish appendicitis from other abdominal conditions. Appendicitis scoring systems, such as the Alvarado score, Appendicitis Inflammatory Response (AIR), and Pediatric Appendicitis Score (PAS), help stratify patients into low-, intermediate-, and high-risk categories, guiding further management. However, their utility is limited, as they rely on non-specific symptoms, physical findings, and basic laboratory parameters (*3*). Imaging techniques such as ultrasound (US) and computed tomography (CT) are considered the current gold standard for diagnosing acute appendicitis. In children, US is typically the gold standard, offering a radiation-free, accessible option with a sensitivity of 99.6% and specificity of 99.0% (*4*). However, its accuracy is highly operator-dependent and can be less effective in patients with a high body mass index or atypical appendix positioning. In adults, CT, with its high sensitivity of 91% and specificity of 90%, is often preferred, although its use is limited by concerns over radiation exposure, high costs, and availability (*5*). These diagnostic limitations contribute to high rates of negative appendectomies, which involve removal of a normal appendix due to misdiagnosis, reported in 19-39% of cases (*6*). The primary cause of this unsatisfactory outcome is the clinical overlap between acute appendicitis and other abdominal conditions, such as mesenteric lymphadenitis, gastroenteritis, gynecologic disorders, and urinary tract infections, which may present with similar signs and symptoms but often do not require surgical intervention (*7*). The highest rate of negative appendectomies is observed in women of reproductive age, often due to pelvic conditions that mimic appendicitis, including tubo-ovarian pathologies, pelvic inflammatory disease, endometriosis, and ectopic pregnancy (*8*). Although the frequency varies across centers, both negative appendectomy and perforated appendicitis are associated with increased morbidity, mortality, and high treatment costs (*9*). This high rate of negative appendectomies is largely attributable to the absence of a single, highly sensitive and specific biological marker for acute appendicitis that is widely accessible. Given these concerns, a simple and cost-effective alternative approach to rule out acute appendicitis would be highly desirable, potentially offering a solution for initial screening. Recent studies have identified proteins with differential expression in appendicitis, indicating inflammation and immune system activity. Leucine-rich alpha-2-glycoprotein 1 (LRG1) and calprotectin have emerged as promising biomarkers, potentially enhancing diagnostic accuracy by providing additional insights in challenging cases (*10*, *11*).

This review aims to evaluate the potential role of LRG1 and calprotectin as diagnostic biomarkers in suspected acute appendicitis, emphasizing the need for further studies to thoroughly assess their diagnostic accuracy.

## Materials and methods

A literature search was conducted to identify studies published from 2000 to the present, focusing on observational studies, clinical trials, and original research assessing the diagnostic accuracy of LRG1 and calprotectin in acute appendicitis. The search was performed in PubMed, Scopus, and Web of Science using keywords such as “leucine-rich alpha-2-glycoprotein 1”, “LRG1”, “LRG-1”, “calprotectin”, “S100A8/A9”, “acute appendicitis”, and “biomarkers”. Only English-language publications were included.

**Figure 1:**
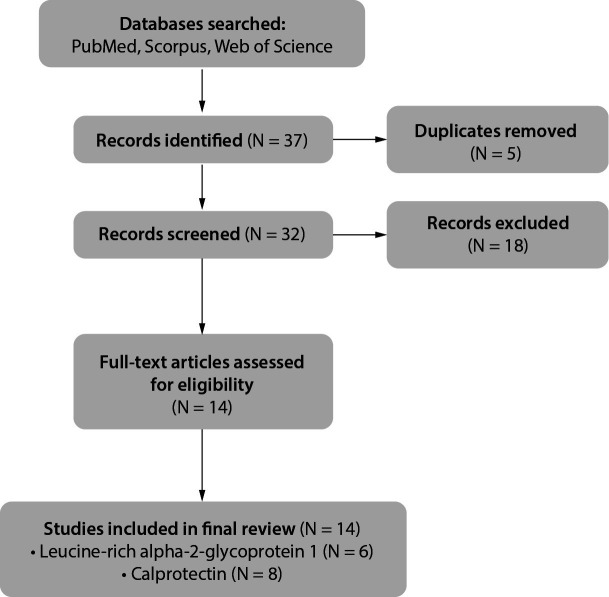
Flow diagram of the study selection process.

Studies were selected if they (*1*) evaluated LRG1 and/or calprotectin as biomarkers for acute appendicitis, (*2*) were peer-reviewed, and (*3*) reported diagnostic metrics. Exclusion criteria included studies that (*1*) did not assess these biomarkers, (*2*) were reviews, case reports, editorials, or conference abstracts, (*3*) lacked sufficient diagnostic data, or (*4*) were not available as free full text. A total of 37 studies were identified, of which 15 met the inclusion criteria. The study selection process is summarised in Figure 1.

Additionally, reference lists of relevant articles were reviewed to identify further studies. While key diagnostic metrics such as sensitivity and specificity were extracted, not all studies provided complete data on predictive values, likelihood ratios, or full ROC analyses. To strengthen the comparability of findings, area under the curve (AUC) values were included whenever available, and cut-off values for biomarker interpretation were extracted where reported.

## Emerging biomarkers in the diagnosis of acute appendicitis

The need for novel biomarkers to complement traditional appendicitis diagnostics is increasing, as standard methods may not always differentiate acute appendicitis from other causes of abdominal pain, especially in atypical cases (*7*, *8*, *12*).

As an acute-phase protein, LRG1 is associated with inflammatory responses, bacterial infections, and neoplastic processes (*13*). Although its precise physiological role remains unclear, LRG1 has been found to participate in processes like cell adhesion and signal transduction (*14*). It has been shown that LRG1 is secreted by differentiating neutrophils, hepatocytes, and mesenteric venules (*15*). Through a proteomic approach, LRG1 has been detected in the blood, urine, and saliva of pediatric patients with acute appendicitis, reflecting its association with the severity of inflammation (*16*, *17*).

Calprotectin (S100A8/A9) is a calcium-binding protein in the S100 family, composed of two subunits: S100A8, known as calgranulin A, and S100A9, known as calgranulin B, which form a heterodimer. These proteins are predominantly found in neutrophils, where calprotectin accounts for up to 60% of the cytoplasmic proteins (*18*). These proteins are crucial in the immune response, particularly in inflammation, where they facilitate chemotaxis and regulate immune cell activation. Its concentrations directly correlate with the extravasation of neutrophils into the intestinal lumen and the histological inflammation of the intestine (*19*). Known for its involvement in gastrointestinal inflammation, calprotectin has become a valuable biomarker in this context (*20*).

## Laboratory determination of leucine-rich alpha-2-glycoprotein 1 and calprotectin in acute appendicitis

Both biomarkers can be measured in different samples, including blood, urine, saliva, and stool (*10*, *11*, *21*, *22*). The LRG1 is primarily measured in blood, typically quantified from serum (*10*). Calprotectin is mainly determined from stool samples, where an extraction procedure is required for stabilisation, but it can also be measured in plasma and, more recently, in serum, where it has gained attention as a potential diagnostic marker (*11*, *22*, *23*).

In clinical practice, obtaining stool samples from emergency department patients may be challenging due to time delays (*22*). Furthermore, saliva collection can be difficult in young children due to the risk of suffocation, inconsistent sample volume, and limited laboratory experience in processing this material, while urine collection may be impractical in uncooperative patients or those with low urine output. Obtaining a urine sample from patients with suspected appendicitis in the emergency department can be difficult, especially since many of these patients are moderately dehydrated. Additionally, the requirement for fasting prior to surgery limits urine production, making it harder to obtain adequate samples for testing. There is also uncertainty about whether creatinine adjustment is needed for accurate measurement of urine samples for this purpose (*24*). Considering the practical limitations of alternative sample types discussed above, blood appears to be the most feasible and commonly used sample for biomarker analysis in patients with suspected acute appendicitis, particularly in emergency settings.

For blood-based measurements, venous blood is collected and placed into vials containing a clot activator for serum separation or an anticoagulant such as ethylenediaminetetraacetic acid (EDTA) for plasma preparation (*10*, *25*). Saliva samples for LRG1 analysis require pre-sampling procedures, such as mouth rinsing to eliminate contaminants, before being collected with swabs (*17*). Blood samples for LRG1 and calprotectin quantification are centrifuged at 1300-2000xg for 10-15 minutes, while saliva samples are centrifuged at 4000xg before being stored at - 80 °C until analysis (*10*, *11*, *17*, *26*). Urine samples were centrifuged at 3000xg for 10 minutes, and stability testing of samples stored at 4 °C for up to 48 hours showed minimal changes in LRG1 concentrations (*21*, *26*). It has been shown that calprotectin remains stable in stool samples at room temperature for up to 7 days, and a sample of less than 5 g is sufficient for reliable measurement (*27*).

Common methods for the determination of LRG1 and calprotectin include enzyme-linked immunosorbent assay (ELISA), immunoturbidimetry, and chemiluminescent immunoassay (CLIA). The most widely used method for both biomarkers is ELISA, due to its good sensitivity, specificity, and cost-effectiveness, but it has a longer turnaround time, typically ranging from a few hours to one day, which limits its use in urgent clinical decision-making (*28*, *29*). To overcome this, quantitative rapid tests based on lateral flow immunochromatography provide results within 15 minutes and are suitable for point-of-care use, though their precision may decrease at higher analyte concentrations (*20*). Immunoturbidimetry enables faster, automated analysis and is increasingly used in routine laboratories, especially for calprotectin, though it may have slightly lower sensitivity than ELISA (*30*). With high sensitivity and rapid, automated processing, CLIA provides reliable results but requires advanced equipment and is less widely available. (*31*).

## Diagnostic value of emerging biomarkers in acute appendicitis

Emerging evidence highlights the diagnostic value of LRG1 and calprotectin in addressing the challenges of early and more accurate detection of acute appendicitis, as summarized in Table 1.

**Table 1:** Diagnostic performance of leucine-rich alpha-2-glycoprotein 1 and calprotectin in acute appendicitis studies

**Study** **(year)**	**Biomarker**	**Biofluid**	**Age** **(years)**	**Participants** **(N)**	**Cut-off^†^**	**Units**	**Sensitivity (%)**	**Specificity (%)**	**AUC value**	**Ref.**
Montero *et al.* (2024)	LRG-1	serum	3-14	200	25.61	ng/mL	91.2	73.2	0.88	(*24*)
Tinor *et al.* (2023)		serum	5-17	92	69.1	µg/mL	100	100	0.98	(*10*)
		saliva			352.6	ng/mL	36	100	0.85	(*17*)
Kakar *et al.* (2021)		serum	7-17	153	51.69	μg/mL	93.8	91.1	0.94	(*16*)
		urine			0.18	μg/mL	54.2	83.9	0.70	
Yap *et al.* (2020)		saliva	4-16	34	0.33	ng/μg	35.5	100	0.77	(*32*)
Salö *et al.* (2016)		urine	3-14	44	0.036	g/mol	86	73	0.86	(*33*)
Kharbanda *et al.* (2012)		serum			40.15	ng/ml	100	35	0.69	
		urine	3-18	176	42	ng/ml	100	23	0.63	(*26*)
		plasma			159	ng/ml	100	27	0.68	
Bealer *et al.* (2010)	CP	plasma	8-76	181	20	‡	93	54	§	(*34*)
Mills *et al.* (2012)		plasma	16-38	848	14	‡	96	16	§	(*35*)
Schellekens *et al.* (2013)		plasma	5-83	233	369	ng/mL	45	83	0.59	(*25*)
Cikot *et al.* (2016)		plasma	19-56	119	46	ng/mL	98.8	83.3	0.92	(*11*)
Sarsu *et al.* (2017)		serum	3-75	120	670	ng/mL	73.3	100	1.00	(*23*)
Hashemy *et al.* (2019)		serum	15-38	149	0.72	mg/dl	70	50	0.58	(*36*)
Zhou *et al.* (2020)		feces	≥ 18	84	106	ug/g	§	§	0.93	(*22*)
The studies included in this table all followed a prospective study design. ^†^Cut-off values indicate the threshold for distinguishing acute appenticitis from the control group. Acute appendicitis was confirmed by histopathological analysis. The composition of the control groups varied depending on the study; some included healthy individuals, while others included patients with abdominal pain in whom appendicitis was excluded through additional diagnostic tests or clinical follow-up. ^‡^Units not defined in text. ^§^The values are not explicity stated in the text. LRG1 - leucine-rich alpha-2-glycoprotein 1. CP - calprotectin. AUC - area under the curve.										

Several studies confirm that serum LRG1 demonstrates high diagnostic performance, with AUC values consistently above 0.88 and specificity often surpassing traditional inflammatory markers such as WBC count and CRP. For example, Tintor *et al.* reported perfect sensitivity and specificity (both 100%) for serum LRG1, while Kakar *et al.* and Montero *et al.* also noted excellent results, supporting its role in emergency diagnostics (*10*, *16*, *24*). However, these findings primarily stem from pediatric populations, and caution is needed when generalizing to adult patients. Differences in immune responses and disease presentation between children and adults may affect biomarker reliability.

Measurement method and sample type significantly influence diagnostic accuracy. While serum LRG1 consistently shows strong performance, alternative sample types such as saliva and urine yield more variable results. Salivary LRG1, as explored by Tintor *et al.* and Yap *et al.*, showed high specificity (100%) but markedly lower sensitivity (35-36%), suggesting limited utility for ruling out appendicitis (*17*, *32*). Urinary LRG1 also demonstrated inconsistent diagnostic accuracy, with AUC values ranging from 0.70 (Kakar *et al.*) to 0.86 (Salö *et al.*), reflecting differences in population size and methodology (*16*, *33*).

In contrast, calprotectin showed higher sensitivity but generally lower specificity. Initial studies by Bealer *et al.* and Mills *et al.* reported sensitivities above 90%, but with specificities below 55%, indicating a tendency for false-positive results - meaning that calprotectin was often elevated in patients without appendicitis, likely due to other inflammatory abdominal conditions, which limits its diagnostic specificity (*34*, *35*).

Schellekens *et al.* reported limited diagnostic accuracy for plasma calprotectin, possibly due to analysis in a mixed-age population and methodological inconsistencies, despite using a relatively high cut-off value (*25*). In contrast, Cikot *et al.* reported better diagnostic performance using a lower cut-off in a pediatric cohort, with more standardized protocols (*11*). These findings suggest that assay performance may be more influenced by population characteristics and laboratory methods than by cut-off value alone. Fecal calprotectin, studied by Zhou *et al.*, achieved an AUC of 0.93, but its practical use in emergency settings is hindered by delays in sample collection and processing (*22*).

Reported diagnostic cut-off values for LRG1 and calprotectin vary notably between studies. For example, serum LRG1 thresholds range from 25.6 ng/mL to 69.1 µg/mL, while calprotectin values span from 14 ng/mL in plasma to 106 µg/g in feces, and additional formats such as ng/µg or g/mol are used in urine and saliva analysis. These inconsistencies complicate interpretation and limit cross-study comparisons. Without harmonized units and clinically validated thresholds, the translation of these biomarkers into routine diagnostic practice remains challenging.

Compared to traditional markers like CRP and WBC, both LRG1 and calprotectin offer improved specificity and sensitivity. Widely used, CRP and WBC lack specificity, as they elevate in various non-appendiceal inflammatory states. In contrast, serum LRG1 provides higher specificity, aiding in reducing false positives, while calprotectin, particularly when measured in plasma or stool, offers greater sensitivity and may help decrease false negatives. Their combined use may enhance diagnostic accuracy, although no studies have yet systematically explored this synergy.

While some studies examined the integration of biomarkers with clinical scoring systems, such as the Pediatric Appendicitis Score (PAS), this area remains underexplored. Yap *et al.* and Salö *et al.* reported improved performance when urinary LRG1 was combined with PAS, suggesting added value in risk stratification (*21*, *33*). However, due to the limited number of such studies, more research is needed to validate these findings across broader populations.

The current review provides a focused synthesis of available studies assessing the diagnostic value of LRG1 and calprotectin in acute appendicitis, including data on different samples, measurement techniques, and cut-off thresholds. A strength of the review lies in its inclusion of both traditional and emerging biomarker data across multiple sample types and its effort to critically evaluate test performance beyond simple enumeration.

However, limitations must be acknowledged. Most of the included studies were conducted in pediatric populations, limiting generalizability to adult patients. Sample sizes were generally small, and the diagnostic protocols and laboratory methods varied widely across studies. Inconsistencies in assay types, reference standards, and reporting of key diagnostic metrics, including cut-off values, reduce comparability. Additionally, the lack of standardized thresholds for biomarker interpretation further complicates clinical application. These limitations underscore the need for larger, multicenter studies using standardized methodologies and including adult patient populations.

## Conclusion

Calprotectin and LRG1 show strong potential as diagnostic biomarkers for acute appendicitis, offering advantages over traditional inflammatory markers. Serum LRG1 demonstrates high specificity, while calprotectin provides strong sensitivity, particularly in plasma and fecal samples.

To overcome current research limitations and strengthen their role in clinical diagnostics, future studies should aim to validate these findings in adult populations, establish standardized measurement methods and cut-off thresholds, and assess combined biomarker use within established clinical scoring systems. These steps may improve diagnostic accuracy, reduce unnecessary imaging and surgical interventions, and support more timely and evidence-based decision-making in emergency settings.

## Data Availability

No data was generated during this study, so data sharing is not applicable to this article.
